# The effect of BeO on heat transfer and durability of nano-CaO-based CO_2_ adsorbents

**DOI:** 10.1039/d1ra09250b

**Published:** 2022-05-04

**Authors:** Hao Liu, Sufang Wu

**Affiliations:** College of Chemical and Biological Engineering, Zhejiang University Hangzhou 310027 China wsf@zju.edu.cn

## Abstract

The solution of decreasing the decomposition temperature of CaCO_3_ and the development of the durability of the CaO-based CO_2_ adsorbent are the key issues in reducing the energy consumption and cost of CO_2_ capture in calcium looping technology. In this work, BeO with high thermal conductivity was chosen as a dopant of the adsorbent to increase the thermal conductivity properties and decomposition properties of CaCO_3_. The endothermic rate of the nano-CaO-BeO/Al_2_O_3_ adsorbent with 15.6 wt% BeO dopant increased by 12.3% compared with that of the nano-CaO/Al_2_O_3_ adsorbent at 720 °C, leading to an increase of 10.1% of CaCO_3_ decomposition rate. The enhancement of the decomposition rate of the nano-CaO-BeO/Al_2_O_3_ adsorbent was significant to lower the regeneration temperature by 50 °C compared with that of the nano-CaO/Al_2_O_3_ adsorbent under calcium looping conditions, which made the total average deactivation rate decrease by 21.0% and made the total residual stable carbonation conversion increase by 27.0% in infinite calcium looping cycles. Strengthening the heat transfer inside the adsorbent material can effectively decrease the regeneration temperature, so as to improve the sorption durability.

## Abstract 2

BeO is firstly doped into nano-CaO/Al_2_O_3_ adsorbents to increase decomposition endothermic heatflow. It will enhance decomposition rate of nano-CaCO_3_, then will reduce decomposition temperature and improve sorption durability of adsorbents.

## Graphical abstract

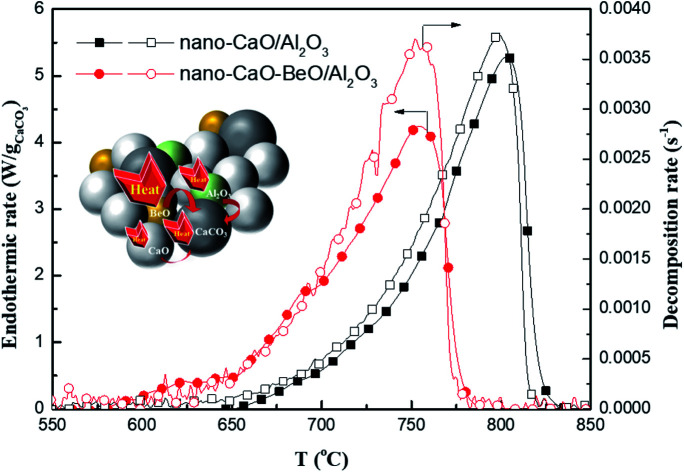

## 1. Introduction

Calcium looping (CaL) technology^1,2^ using CaO carbonation/CaCO_3_ decomposition reversible reactions of the CaO-based adsorbent (eqn (1)) has been widely investigated to reduce greenhouse gas CO_2_ emission in industrial CO_2_ capture.^3–5^ One of the bottleneck problems of CaL technology is that the sorption capacity of the CaO-based adsorbent rapidly decays after several CaL cycles owing to high-temperature sintering,^6–8^ which seriously affects its reversibility and economic feasibility.^9^

1 CaO + CO_2_ ⇔ CaCO_3_, Δ*H*_298_ = −178.8 kJ mol^−1^

In order to obtain complete decomposition of CaCO_3_, a high temperature of around 800–1000 °C is adopted in the regeneration step because of the strong endothermic process and the equilibrium of the CaCO_3_ decomposition reaction.^6^ High regeneration temperature will accelerate the thermal grain growth rate of nano-CaCO_3_ which is the root cause of the deactivation of the nano-CaO-based adsorbent in the CaL process.^10^ Therefore, reducing the regeneration temperature as much as possible will be a crucial issue for improving the durability of the adsorbent and saving calcination energy. Research on reducing the regeneration temperature of the adsorbent by increasing its CaCO_3_ decomposition rate has been carried out, including decreasing the reaction activation energy as well as enhancing mass transfer and heat transfer.

Firstly, the methods of decreasing the activity energy of CaCO_3_ decomposition reaction included introducing steam into the calciner^11–13^ and using nano-CaCO_3_ precursor.^6,14–16^ Valverde *et al.*^17^ found that if the regeneration atmosphere contained steam, the regeneration temperature of limestone would reduce by nearly 50 °C. However, it was found that the existence of steam could also accelerate the sintering of adsorbent at high temperature.^11,13^ On the other hand, nano-CaCO_3_ precursor could significantly reduce the regeneration temperature by nearly 50 °C because of higher specific surface energy,^6^ but the current regeneration temperature of 800 °C still led to the unavoidable deactivation problem of nano-CaO-based adsorbents.

Besides, researches about mass transfer enhancement for developing the CaCO_3_ decomposition rate were mainly focused on increasing the porosity of the adsorbent.^18–24^ Campbell *et al.*^18^ found that when the porosity of CaCO_3_ increased from 0.33 to 0.66, the regeneration time could be shortened from 30 min to 15 min. However, the porosity of the adsorbent would gradually decrease as the CaL cycles increasing,^20^ which would lead to the loss of enhancement effect on the mass transfer, so as to the decrease of the CaCO_3_ decomposition rate. Recent studies on heat transfer enhancement were mainly focused on increasing the external heating rate of the calciner,^25^ including using high thermal conductivity sweep gas He^26^ and microwave irradiation.^27^ But the development of the CaCO_3_ decomposition rate by using the new method was not obvious.

Since the effect of external heat transfer enhancement was limited, better CaCO_3_ decomposition rate improvement might be obtained by enhancing internal heat transfer performance of material. Because the decomposition of CaCO_3_ belonged to a kind of strong endothermic reaction (Δ*H*_298_ = 178.8 kJ mol^−1^). The adsorbent with higher average thermal conductivity could get more heat in unit time for CaCO_3_ decomposition reaction,^28^ which had been seldom studied in detail. In this work, it was proposed that the introduction of the dopant with higher thermal conductivity into the adsorbent might increase the endothermic rate leading to a higher CaCO_3_ decomposition rate. Within the regeneration temperature range of 730–830 °C, BeO (44.2 W m^−1^ K^−1^) had significantly higher average thermal conductivity than those of CaO (7.0 W m^−1^ K^−1^), CaCO_3_ (0.17 W m^−1^ K^−1^) and carrier Al_2_O_3_ (7.5 W m^−1^ K^−1^).^29^

In this study, samples were obtained by varying BeO dopant contents of nano-CaO-based adsorbents. The composition, pore structure and morphology of samples were characterized for eliminating the effect of mass transfer on the CaCO_3_ decomposition rate. After that, the enhancement effect of BeO dopant with high thermal conductivity on the CaCO_3_ endothermic rate was tested. Then, the relationship between heat transfer performance and CaCO_3_ decomposition rate was systematically investigated. Finally, the reduction of regeneration temperature of the nano-CaO-BeO/Al_2_O_3_ adsorbent was tested for the improvement of its cyclic sorption durability.

## 2. Experimental

### 2.1 Preparation of nano-CaO-based adsorbents

A certain proportion of nano-CaCO_3_ powder (>95% purity, 70 nm, Hu Zhou Ling Hua Ltd China) and dopant BeO (>99% purity, Aladdin) were added into 50 vol% ethanol aqueous solution under ultrasonic dispersion. Then the aluminum sol (10 wt%, Zibo Longao Ltd) was added to this slurry and stirred at 80 °C. The weight fraction of carrier Al_2_O_3_ was set as 15 wt% in the nano-CaO-based adsorbent. The slurry was allowed to dry overnight at 110 °C. Finally, the resulting sample went through calcination at 500 °C for 3 h under N_2_ atmosphere for dehydration. All adsorbent powders were ground to be 60–80 μm sized particles before being tested further.

The designation and composition of adsorbents are listed in Table 1. Ca/Al represented the nano-CaO/Al_2_O_3_ adsorbent without any dopants. CaBe*α*/Al (*α* = 0.125–4) represented nano-CaO-BeO/Al_2_O_3_ adsorbent doped with BeO and molar ratio of CaO to BeO was *α*.

**Table 1:** Nano-CaO-based adsorbents' composition and designation

No.	Sample	Molar ratio of CaO to BeO dopants	CaO content (wt%)	BeO dopant content (wt%)
1	Ca/Al	—	85.0	0
2	CaBe4/Al	4 : 1	76.2	8.8
3	CaBe2/Al	2 : 1	69.4	15.6
4	CaBe1/Al	1 : 1	58.8	26.2
5	CaBe0.5/Al	1 : 2	44.8	40.2
6	CaBe0.25/Al	1 : 4	30.5	54.5
7	CaBe0.125/Al	1 : 8	18.6	66.4

### 2.2 Test and calculation method

The average thermal conductivity of the adsorbent at high temperature (730 °C–830 °C) could not be tested directly because of the occurrence of CaCO_3_ decomposition reaction. Under the same test condition, there was a positive correlation between the endothermic rate and the thermal conductivity of the same material.^28^ Therefore, the endothermic rate test value of the adsorbent could be used to represent its heat transfer performance. Endothermic rates of adsorbents with varied BeO contents during continuous heating process were measured by simultaneous thermal analyzer (TGA/DSC3+, METTLER TOLEDO). According to operation conditions of the CaL process, the test range of temperature was set as 500–900 °C with heating rate of 15 °C min^−1^ and the test atmosphere was N_2_. Weight loss (CO_2_ desorption) values of adsorbents were also measured during heating process. The decomposition conversion and decomposition rate were calculated as eqn (2) and (3).^30^2
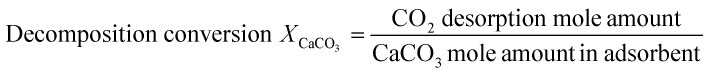
3
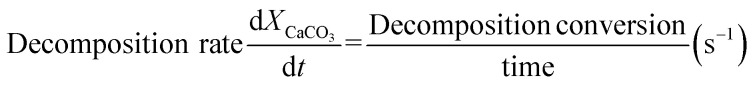


**Fig. 1:**
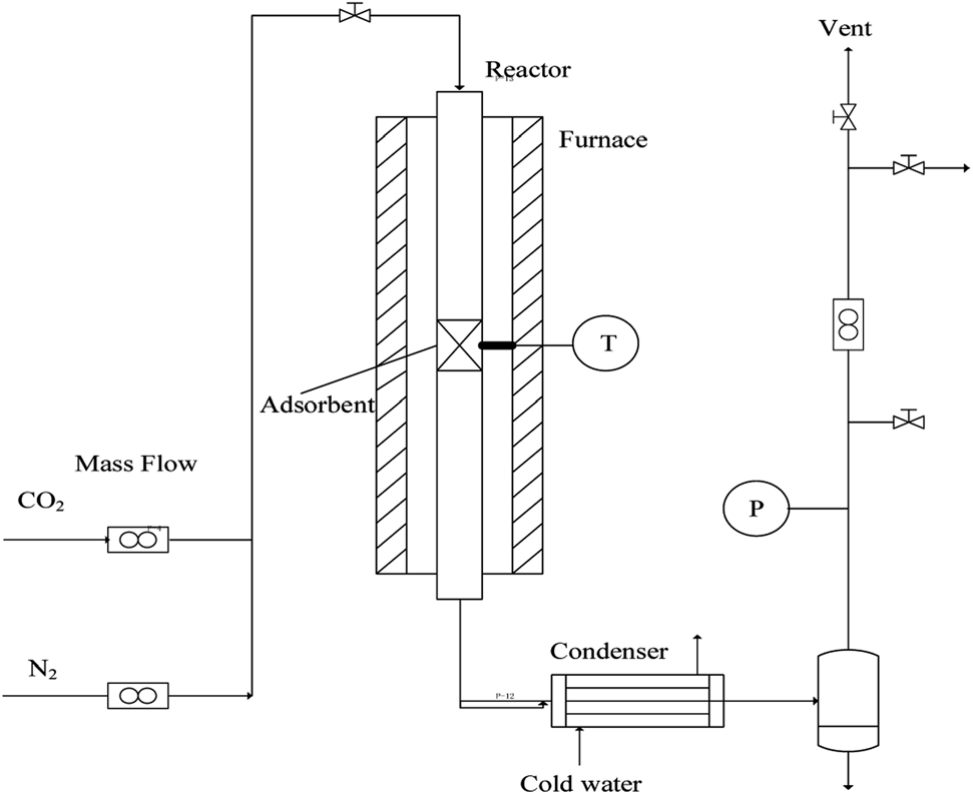
Diagram of the fixed bed reactor system.

In order to evaluate the cyclic sorption performance, the nano-CaO-based adsorbent went through the CaL process in the laboratory fixed bed reactor. The diagram of the reactor is shown in Fig. 1, whose diameter was 12 mm × 1 mm and length was 400 mm. The temperature was controlled by a programmable heating furnace and the feed flowrate of CO_2_ and N_2_ were controlled by the mass flowmeter. Before each test, 2.0 g adsorbent was loaded in the center of fixed bed reactor. Firstly, the reactor was heated to the specified regeneration temperature with heating rate of 15 °C min^−1^. The regeneration temperature was set as 800 °C, 770 °C, 750 °C or 730 °C and the regeneration atmosphere was pure N_2_ atmosphere. The regeneration time was set according to the complete conversion time of CaCO_3_ decomposition under specific regeneration temperature. When regeneration process was finished, temperature of reactor would be cooled down to carry out the carbonation process. The carbonation temperature was set as 600 °C. The carbonation atmosphere and time were fixed as 20 vol.% CO_2_/80 vol.% N_2_ and 20 min, respectively. After carbonation, the temperature of reactor would return to the regeneration temperature to form a complete CaL cycle. The sorption capacity and carbonation conversion of adsorbents were calculated according to eqn (4) and (5).^31^4
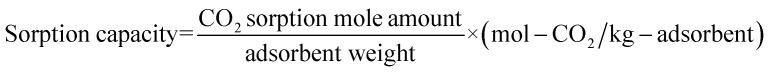
5
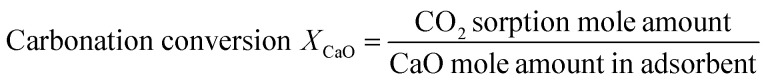


In order to predict the residual stable carbonation conversion of adsorbents after infinite CaL cycles and compare the average deactivation rate of different adsorbents, researchers established some semi-empirical equations to fitting the number of cycles *N* and corresponding carbonation conversion *X*_*N*_ using test results of carbonation conversion during initial finite CaL cycles, which were deactivation mathematical models of adsorbents.^32–34^ Among those, the deactivation mathematical models proposed by Arias *et al.*^34^ owned the best fitting accuracy for limestone adsorbents, as shown in eqn (6). Therefore, eqn (6) was considered to be used to evaluate the durability of nano-CaO-based adsorbents with some necessary adjustments in this work.6
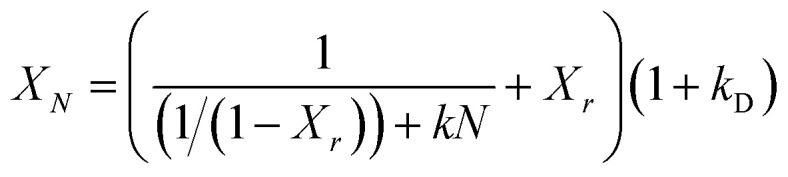


The carbonation process of the CaO-based adsorbent could be divided into fast section and slow section. In eqn (6), *k* and *X*_*r*_ represented the average deactivation rate and the residual stable carbonation conversion in the fast section. *k*_D_ represented the contribution of the slow section to deactivation comparing to the fast section.^34^ For the micro-CaO-based adsorbent, *k*_D_ was so small that *k* could be used to represent the average deactivation rate of adsorbents approximately. However, it was found that for the nano-CaO-based adsorbent, the effect of slow section on the deactivation could not be ignored in this work. Therefore, new parameters were needed to take into account of both fast and slow sections. *k*_*t*_ = *k*/(1 + *k*_D_) represented the total average deactivation rate of the adsorbent (total *k*) and *X*_*r*−*t*_ = (1 + *k*_D_)*X*_*r*_ represented the total residual stable carbonation conversion of the adsorbent (total *X*_*r*_). In addition, *c* = 1/[(1 + *k*_D_)(1 − *X*_*r*_)]. Then eqn (6) would be converted into eqn (7), which could be more simple and clear to reflect the deactivation performance of nano-CaO-based adsorbents.7
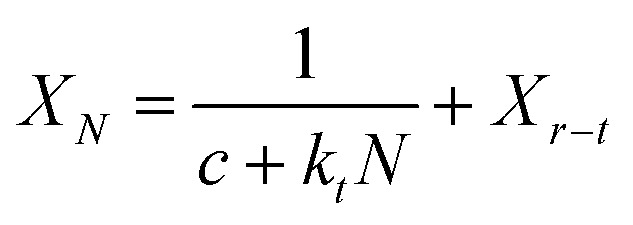


### 2.3 Characterization of microstructure

Crystalline phases of components of the adsorbent were determined by X-ray diffraction (XRD: D/MAX-RA, Rigaku, Japan). Specific surface area (BET method) and desorption average pore diameter (BJH model) analyses were conducted by nitrogen physisorption at −196 °C by means of a BEL SORP-mini II apparatus. The morphology of the adsorbent was investigated by scanning electron microscopy (SEM, S-4800, Hitachi, Japan).

## 3. Results and discussion

### 3.1 Effect of BeO dopant on mass and heat transfer of adsorbents

**Fig. 2:**
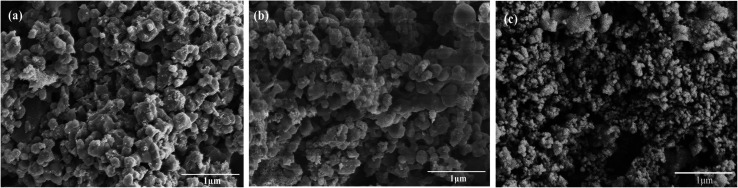
SEM images of (a) Ca/Al, (b) CaBe4/Al, (c) CaBe0.125/Al.

In order to explore the influence of doping BeO on the mass transfer performance of the nano-CaO-based adsorbent, the microstructure of adsorbents including morphology, pore structure and crystal phase were measured firstly. Fig. 2(a)–(c) are SEM pictures of Ca/Al adsorbent, CaBe4/Al adsorbent and CaBe0.125/Al adsorbent, respectively. It could be seen that comparing to Ca/Al adsorbent, there were some smaller BeO grains on the surface of calcite grains in CaBe4/Al adsorbent, and the number of BeO grains increased significantly in the CaBe0.125/Al adsorbent. It showed that the morphology of nano-CaCO_3_ grains were not affected by BeO grains under simple microscopic physical mixing, which could not improve the mass transfer performance of the adsorbent.

Mean pore diameters of adsorbents are listed in Table 2. As the doping content of BeO rose up, the mean pore diameter of the adsorbent decreased obviously (from 34.5 nm of Ca/Al adsorbent to 27.5 nm of CaBe0.125/Al adsorbent). This was due to the increase of BeO content with smaller size, which leaded to smaller mean pore diameter of the adsorbent. The decrease of mean pore diameter of the adsorbent would limit the internal diffusion of CO_2_,^20^ which had a negative impact on the mass transfer and regeneration rate.

**Table 2:** The mean pore diameter of nano-CaO-based adsorbents

Sample	Mean pore diameter (nm)
Ca/Al	34.5
CaBe4/Al	33.7
CaBe2/Al	33.9
CaBe1/Al	31.4
CaBe0.5/Al	30.5
CaBe0.25/Al	31.0
CaBe0.125/Al	27.5

**Fig. 3:**
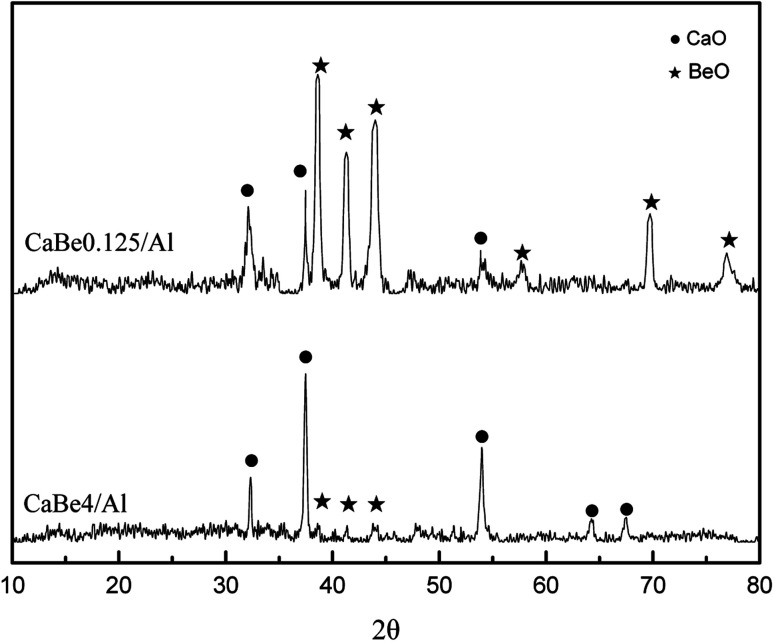
X-ray diffractograms of CaBe4/Al and CaBe0.125/Al adsorbents after decomposition.

XRD test results of CaBe4/Al adsorbent and CaBe0.125/Al adsorbent after regeneration are shown in Fig. 3. Characteristic peaks of CaO and BeO could be found in both adsorbents and BeO peaks were more obvious for CaBe0.125/Al adsorbent because of more BeO contents. Characteristic peaks of Al_2_O_3_ could not be observed in both adsorbents due to its amorphous crystal phase.^35^ In addition, there were no solid-phase reactions between BeO and CaO or between BeO and Al_2_O_3_ found in both adsorbents, which indicated that BeO was a kind of completely inert dopant. In fact, the temperature of more than 1300 °C was required for the solid-phase reaction between BeO and Al_2_O_3_,^36^ which was much higher than regeneration temperature of 800 °C in CaL process.^6^ In conclusion, the introduction of BeO would not improve the mass transfer performance of adsorbents owing to the microstructure characterization.

**Fig. 4:**
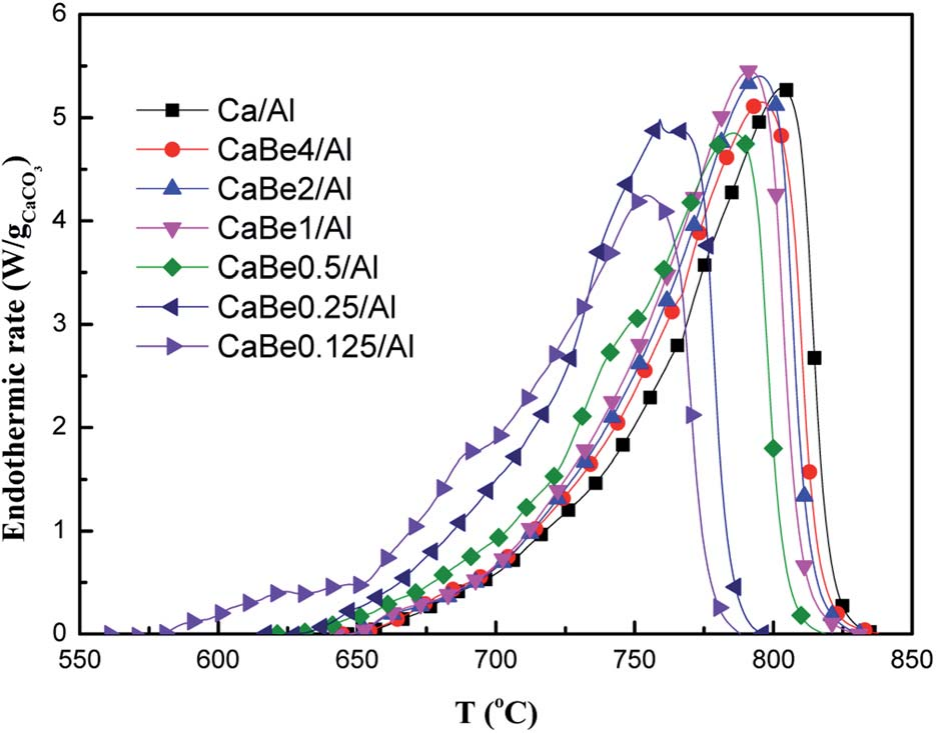
Comparison of endothermic rates of nano-CaO-based adsorbents with different BeO doping contents during continuous heating process.

Furthermore, the effect of high thermal conductivity BeO doping on the heat transfer performance of adsorbents was studied. Endothermic rates of CaCO_3_ decomposition in adsorbents with different BeO contents during continuous heating process were tested to compare the heat transfer performance, which are shown in Fig. 4. According to the inflection point of the curve, the endothermic rate curve could be divided into three temperature sections including low temperature section (<650 °C), medium temperature section (650–800 °C) and high temperature section (>800 °C), which corresponded to three stages of CaCO_3_ decomposition reaction in heating process. In the low temperature section, the temperature was too low for CaCO_3_ to start the decomposition reaction leading to the small endothermic rate. As the temperature rose up to the medium temperature section, the endothermic rate of the adsorbent increased dramatically, corresponding to CaCO_3_ decomposition occurrence. Finally, when temperature reached high temperature section, the endothermic rate of the adsorbent decreased to zero, indicating the end of the CaCO_3_ decomposition reaction.

Among these temperature sections, there were some differences of the effect of BeO doping on heat transfer performance of the adsorbent. When temperature was 650 °C, the endothermic rate of CaBe0.125/Al adsorbent, CaBe4/Al adsorbent and Ca/Al adsorbent were 0.48 W g_CaCO_3__^−1^, 0.04 W g_CaCO_3__^−1^ and 0.02 W g_CaCO_3__^−1^ respectively with little difference. When temperature was 720 °C, the endothermic rate of CaBe0.125/Al adsorbent, CaBe4/Al adsorbent and Ca/Al adsorbent were 2.66 W g_CaCO_3__^−1^, 1.19 W g_CaCO_3__^−1^ and 1.06 W g_CaCO_3__^−1^ respectively, which indicated that BeO doping could enhance heat transfer performance of the adsorbent. It was also found that as BeO doping contents increased, the endothermic rate improved obviously. BeO (44.2 W m^−1^ K^−1^) had significantly higher average thermal conductivity than those of CaO (7.0 W m^−1^ K^−1^), CaCO_3_ (0.17 W m^−1^ K^−1^) and carrier Al_2_O_3_ (7.5 W m^−1^ K^−1^) in the regeneration temperature range of 730–830 °C.^29^ According to Fourier's law, the nano-CaO-BeO/Al_2_O_3_ adsorbent with higher average thermal conductivity could get more heat in unit time for strong endothermic CaCO_3_ decomposition reaction (Δ*H*_298_ = 178.8 kJ mol^−1^).^28^ The discussion of high temperature section was unnecessary because the temperature was too high and the decomposition reaction had finished. It was concluded that the enhancement effect of BeO with high thermal conductivity could be reflected on the heat transfer performance of the adsorbent and the optimal temperature range was in the medium temperature section.

### 3.2 Effect of heat transfer enhancement on CaCO_3_ decomposition rate

**Fig. 5:**
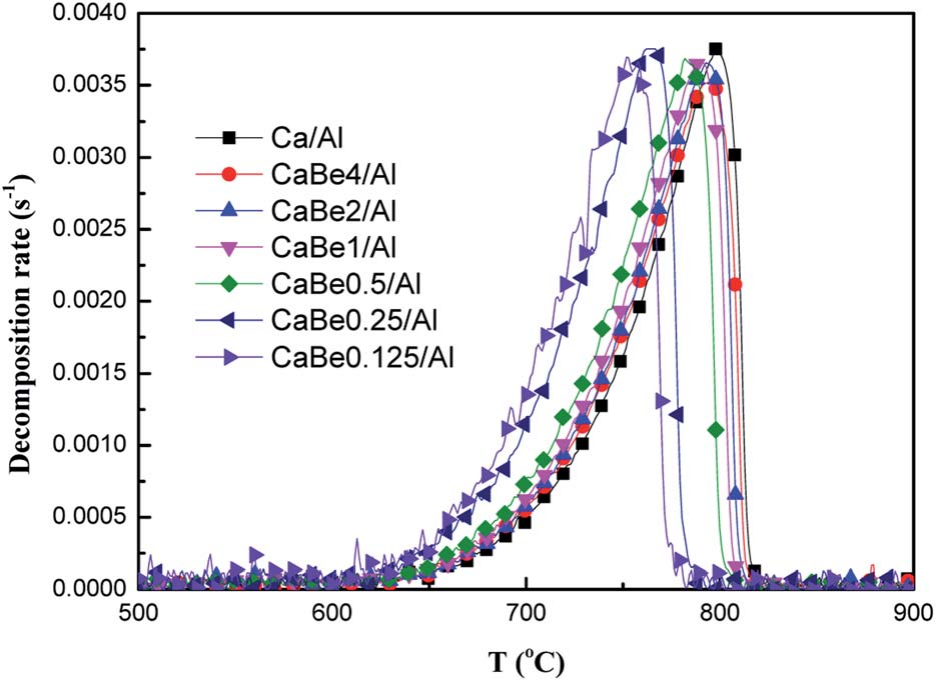
Comparison of decomposition rates of CaCO_3_ in nano-CaO-based adsorbents with different BeO doping contents during continuous heating process.

Subsequently, it was necessary to explore the effect of heat transfer enhancement of adsorbents on the CaCO_3_ decomposition rate. Experimental results of CaCO_3_ decomposition rates of nano-CaO-based adsorbents with different BeO doping contents during continuous heating were compared, as shown in Fig. 5. It could be seen that the configuration of the decomposition rate curve was consistent with that of the endothermic rate curve, which meant that the decomposition rate was actually controlled by the endothermic rate of the adsorbent. Similarly, the effective enhancement of decomposition rate mainly occurred in the medium temperature section of 650–800 °C. When temperature was 720 °C, decomposition rate of CaBe0.125/Al adsorbent, CaBe4/Al adsorbent and Ca/Al adsorbent were 2.1 × 10^−3^ s^−1^, 9.3 × 10^−4^ s^−1^ and 8.4 × 10^−4^ s^−1^ respectively. It should be noted that as the endothermic rate of CaCO_3_ in adsorbents increased, faster CaCO_3_ decomposition rates would be got at the same temperature.

According to the results in Fig. 5, the related characteristic temperatures of decomposition rates in adsorbents are summarized in Table 3. The initial temperature (*T*_di_), the maximum temperature (*T*_dm_) and the final temperature (*T*_df_) of decomposition reaction represented the characteristic temperatures when the decomposition rate started to be positive values, reached maximum values, and returned to values of zero, respectively. As shown in Table 3, *T*_di_, *T*_dm_ and *T*_df_ of CaBe0.125/Al adsorbent were 92 °C, 46 °C and 39 °C lower than those of Ca/Al adsorbent respectively. It could be found that when the decomposition rate at lower temperature decreased, the whole peak of decomposition rate curve would move toward lower temperature direction (Fig. 5), which meant that the regeneration temperature of the adsorbent was reduced successfully.

**Table 3:** The decomposition characteristic temperatures of adsorbents with different BeO doping contents

Sample	*T*_di_ (°C)	*T*_dm_ (°C)	*T*_df_ (°C)
Ca/Al	651	798	820
CaBe4/Al	647	794	816
CaBe2/Al	639	793	813
CaBe1/Al	637	791	810
CaBe0.5/Al	623	782	806
CaBe0.25/Al	613	762	787
CaBe0.125/Al	559	752	781

### 3.3 Effect of regeneration temperature decline of adsorbents in CaL process

It was necessary for nano-CaO-BeO/Al_2_O_3_ adsorbent to own enough sorption capacity for usage of CaL process. Therefore, the sorption capacity and carbonation conversion of adsorbents with different BeO doping contents were compared firstly and the test results are shown in Table 4. Carbonation step was operated at 600 °C with 20 vol% CO_2_/80 vol% N_2_ for 20 min and regeneration step was operated at 800 °C with pure N_2_ for 50 min. Increasing the doping amount of BeO could improve the heat transfer enhancement effect, but the overall sorption capacity and the carbonation conversion would be both reduced. Because the active CaO content was reduced and the average pore size decreased leading to CO_2_ mass transfer limitation of carbonation process. CaBe2/Al adsorbent was chosen as the follow-up research sample owing to its adequate sorption capacity (9.9 mol kg^−1^) and carbonation conversion (80.3%).

**Table 4:** The sorption capacity and carbonation conversion of adsorbents with different BeO doping contents

Sample	Sorption capacity (mol kg^−1^)	*X*_*N*_
CaBe4/Al	11.7	0.859
CaBe2/Al	9.9	0.803
CaBe1/Al	8.1	0.773
CaBe0.5/Al	5.9	0.736
CaBe0.25/Al	4.3	0.796
CaBe0.125/Al	2.8	0.844

It was needed to be explored whether heat transfer enhancement of the adsorbent by BeO dopant could reduce the constant regeneration temperature during CaL process. The test results of regeneration time and decomposition conversion of CaAl adsorbent calcined at 800 °C and 770 °C as well as CaBe2Al adsorbent regenerated at 800 °C, 770 °C, 750 °C and 730 °C are listed in Table 5. The regeneration time referred to the time required for the CaCO_3_ decomposition rate decreasing to zero. The results showed that when the regeneration temperature of Ca/Al adsorbent reduced from 800 °C to 770 °C, the regeneration time would be extended from 46 min to 83 min and the decomposition conversion would be reduced from 97.7% to 83.4%. Therefore, in order to ensure the nearly complete decomposition of CaCO_3_, the minimum regeneration temperature of Ca/Al adsorbent was 800 °C. In contrast, the CaCO_3_ decomposition rate of CaBe2/Al adsorbent could be significantly improved at lower regeneration temperature. At 750 °C, the regeneration time of CaBe2/Al adsorbent was only 48 min, and its decomposition conversion was still as high as 95.1%. However, its regeneration time would greatly increase to 72 min and decomposition conversion would significantly reduce to 85.3% at lower 730 °C. It could be concluded that the minimum regeneration temperature of CaBe2/Al adsorbent was 750 °C, which meant that the heat transfer enhancement of the adsorbent could effectively reduce the regeneration temperature of 50 °C in CaL process.

**Table 5:** Regeneration properties of Ca/Al and CaBe2/Al adsorbents

Sample	Regeneration temperature (°C)	Regeneration finished time (min)	Decomposition conversion
Ca/Al	800	46	0.977
Ca/Al	770	83	0.834
CaBe2/Al	800	37	0.964
CaBe2/Al	770	42	0.958
CaBe2/Al	750	48	0.951
CaBe2/Al	730	72	0.853

### 3.4 Sorption durability improvement of adsorbents in CaL process

**Fig. 6:**
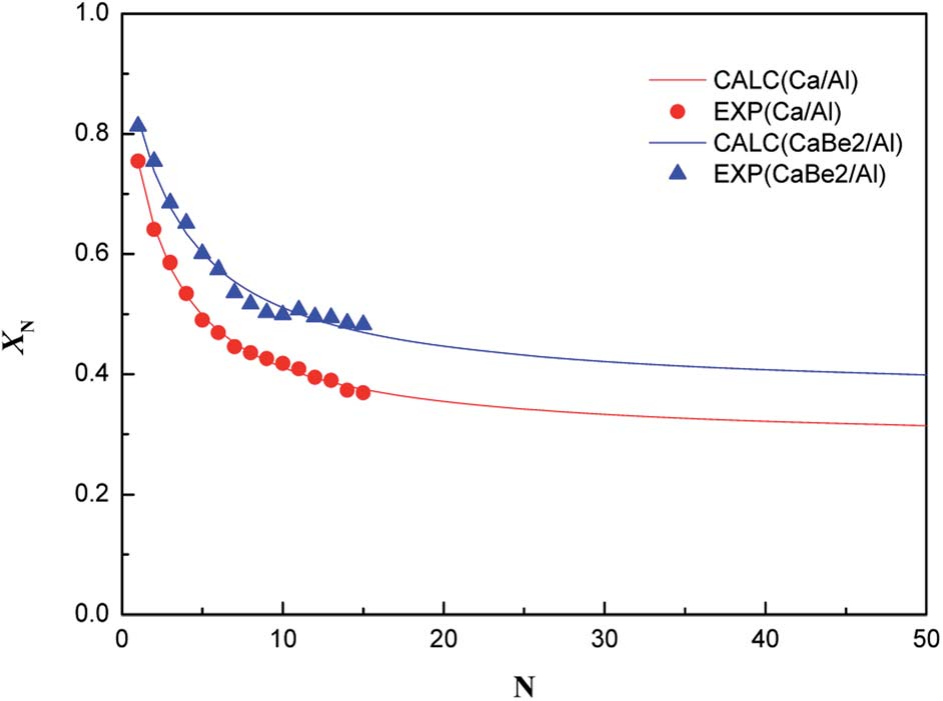
Experimental points and calculation fitting curves of cyclic carbonation conversion of CaBe2/Al and Ca/Al adsorbents (EXP: experimental results, CALC: calculated results).

Finally, in order to explore the effect of regeneration heat transfer enhancement on sorption durability of the nano-CaO-based adsorbent in CaL process, the evolution of carbonation conversion of CaBe2/Al and Ca/Al adsorbents during 15 CaL cycles were tested in the fixed bed reactor. The results are shown in Fig. 6. The carbonation conditions of two samples were both set as 600 °C-20 vol.% CO_2_/80 vol.% N_2_-20 min. The regeneration condition of CaBe2/Al adsorbent was set as 750 °C-100 vol.% N_2_-50 min, and the regeneration condition of Ca/Al adsorbent was set as 800 °C-100 vol.% N_2_-50 min.

It could be found in Fig. 6 that the carbonation conversion (sorption capacity) of CaBe2/Al adsorbent gradually decreased from 81.3% (10.0 mol kg^−1^) to 48.3% (5.9 mol kg^−1^) in 15 CaL cycles, which lost 40.6% of initial carbonation conversion. The carbonation conversion (sorption capacity) of Ca/Al adsorbent decreased from 75.5% (11.4 mol kg^−1^) to 36.9% (5.6 mol kg^−1^) in 15 CaL cycles, which lost 51.1% of initial carbonation conversion. The deactivation mathematical models of CaBe2/Al and Ca/Al adsorbents were fitted based on the experimental data and eqn (7), as shown in eqn (8) and (9). The average deviation between experimental results and calculated values of carbonation conversion of CaBe2/Al and Ca/Al in 15 CaL cycles were only 2.0% and 0.3% respectively, indicating that the calculated values were consistent with the experimental results. According to the equations, the *k*_*t*_ of CaBe2/Al (0.505) was lower than that of Ca/Al (0.639), while the *X*_*r*−*t*_ of CaBe2/Al (0.362) was higher than that of Ca/Al (0.285). CaBe2/Al adsorbent with heat transfer enhancement had lower regeneration temperature so that the sintering degree of the adsorbent in regeneration step was reduced, leading to a better sorption durability and a higher residual stable carbonation conversion.8
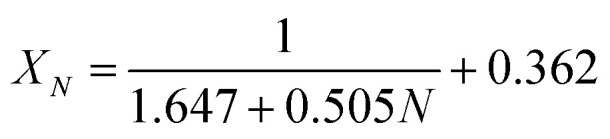
9
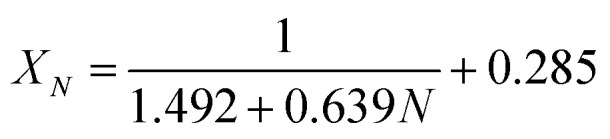


Compared with the results of other researches on improving the cyclic durability of CaO-based adsorbents through enhancing decomposition of CaCO_3_, the introduction of high thermal conductivity BeO could get more obvious improvement. Li Z. H. *et al.*^37^ introduced steam into the regeneration atmosphere of limestone, which would increase the CaCO_3_ decomposition rate of 1.3% min^−1^. The loss ratio of initial carbonation conversion was decreased for 2.4% through 17 CaL cycles. Lu S. Q. *et al.*^20^ increased the mean pore diameter of nano-CaO-Al_2_O_3_ adsorbents from 19 nm to 54 nm, which increased the CaCO_3_ decomposition rate by 1% min^−1^ and decreased the regeneration temperature by 25 °C. The loss ratio of initial carbonation conversion was reduced for 8% through 10 CaL cycles. In contrast, doping high thermal conductivity BeO could reduce the regeneration temperature by 50 °C (from 800 °C to 750 °C), and the loss ratio of initial carbonation conversion was reduced for 10.5% through 15 CaL cycles, which showed best technical advantage.

## 4. Conclusions

In this work, high thermal conductivity BeO with different doping contents 8.8–66.4 wt% was added into the nano-CaO-based adsorbent to study its heat transfer enhancement effect of CaCO_3_ decomposition and the improvement of the cyclic sorption durability. In order to obtain adequate sorption capacity (higher than 10 mol kg^−1^), 15.4 wt% BeO doping amount was suitable. The existence of 15.6 wt% BeO dopants could effectively develop the endothermic rate of CaCO_3_ decomposition in adsorbents by 12.3%, leading to an increase of 10.1% of CaCO_3_ decomposition rate regenerated at the temperature of 720 °C. The promotion of decomposition rate of CaCO_3_ at lower temperature was the reason for the decrease of decomposition temperature of nearly 50 °C, which could bring lower sintering degree and better sorption durability of adsorbents in CaL process with the decrease of total average deactivation rate decrease of 21.0% and the increase of total residual stable carbonation conversion increase of 27.0% in infinite calcium looping cycles.

## Conflicts of interest

There are no conflicts to declare.

## Supplementary Material
